# ﻿An analytical synopsis of caddisfly (Insecta, Trichoptera) taxonomic history and progress in Canada and the United States

**DOI:** 10.3897/zookeys.1263.147986

**Published:** 2025-12-10

**Authors:** Phillip N. Hogan, David C. Houghton, Kelly Murray-Stoker, R. Edward DeWalt, Andrew K. Rasmussen, John C. Morse

**Affiliations:** 1 Department of Entomology, University of Illinois, Urbana-Champaign, Urbana, IL 61801, USA University of Illinois Urbana-Champaign United States of America; 2 Department of Biology, Hilldale College, Hillsdale, MI 49242, USA Hilldale College Hillsdale United States of America; 3 Department of Natural Sciences and Mathematics, Oxford College of Emory University, Oxford, GA 30054, USA Oxford College of Emory University Oxford United States of America; 4 Illinios Natural History Survey, Champaign, IL, 61820 USA Illinios Natural History Survey Champaign United States of America; 5 Center for Water Resources, Florida A&M University, Tallahassee, FL, 32307 USA Florida A&M University Tallahassee United States of America; 6 Department of Plant & Environmental Sciences, Clemson University, Clemson, SC, 29634 USA Clemson University Clemson United States of America

**Keywords:** Aquatic insects, faunistic, North America, species checklist

## Abstract

A total of 1,510 caddisfly species representing 28 families and 155 genera are reported from the 63 states and provinces of Canada and the United States of America (USA). These species have been described over a period of nearly 270 years, with the most prolific period occurring during the 1930–1940s. The families Hydroptilidae (307), Limnephilidae (255), and Hydropsychidae (159) contain the most species, whereas six families contain less than five species each. Canada and the USA host 644 and 1,487 species, respectively. The states and provinces with the greatest species richness are Tennessee (384), Virginia (383), and Alabama (378), and those with the least are Rhode Island (27), Prince Edward Island (23), and Nunavut (15). Differences in state species assemblages largely followed a geographic pattern, with a non-metric multidimensional scaling ordination suggesting six regions of caddisfly diversity corresponding to the central, far north, northeastern, northwestern, southeastern, and southwestern portions of the study area. Caddisfly species richness was highest in the southeastern region, despite being the smallest region of the six, and lowest in the far north. Species rarefaction predicted 129–181 species remain to be discovered within the two countries, while multiple linear regression modeling using common environmental variables suggested 17 states and provinces with at least 50 species remaining to be found in each.

## ﻿Introduction

Trichoptera represents the most species-rich order of primarily aquatic insects, with the number of known, extant species approaching 17,300 in 52 families globally (Morse et al. 2019; Morse 2025). Of the orders containing aquatic taxa, only Diptera contains more freshwater species (Adler and Courtney 2019). Caddisflies are present on all continents except Antarctica. The proliferation of caddisfly diversity is, in part, the result of inhabiting a wide variety of freshwater habitat types and is tied to the evolution of portable case-making behavior and other uses of labial silk, with subsequent exploitation of many habitat types (Mackay and Wiggins 1979; Wiggins 1996; Frandsen et al. 2024).

Caddisflies perform many ecologically important services within freshwater ecosystems, made even more valuable by the large proportion of invertebrate macrofaunal biomass that caddisflies represent (Morse et al. 2019). Larvae contribute greatly to the cycle and transfer of carbon and nutrients through the processing of fine and coarse particulate organic matter, herbivory of plant tissue, and predation upon other aquatic macroinvertebrates. Adult dispersal from their natal aquatic habitats into the riparian region of terrestrial systems links habitats in food webs through the transfer of organic material, and nutrients (Hicks et al. 2005; Winder et al. 2005; Francis et al. 2006). Species that spin and maintain silken nets (e.g. Annulipalpia) can be very abundant within stream reaches (Wallace and Merritt 1980), leading to the consolidation of sediments within stream beds and supporting habitat stability for other organisms (Cardinale et al. 2004). Further, the diversity of functional feeding groups and robust response to anthropogenic disturbances across different habitat types promotes use of caddisfly assemblages in water quality biomonitoring (Resh and Unzicker 1975; Lenat 1988; Dohet 2002; Houghton 2025).

Projections of global caddisfly richness estimate that only 20–25% of species have been described, with most undescribed taxa occurring in the Neotropical, Oriental, and Palearctic regions (de Moor and Ivanov 2008). The Neotropical and Oriental regions contain the highest known species richness (Morse et al. 2019) and also the highest rates of species descriptions during the past four decades (Morse 2016; Santos et al. 2020). In contrast, the rate of caddisfly species descriptions over the same period appears to have declined within the Nearctic and eastern Palearctic regions (Wiberg-Larsen 2008).

**Figure 1.**
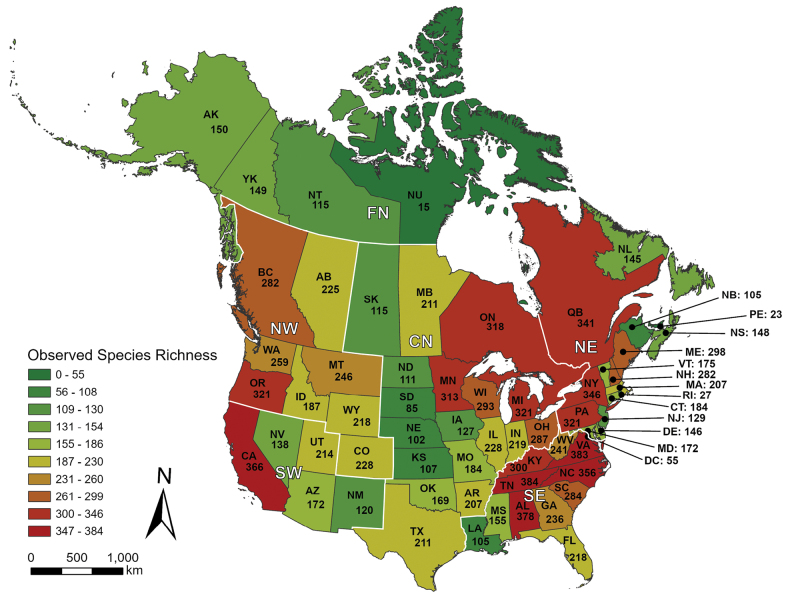
The 63 political units (states and provinces) composing continental Canada and the USA, showing the number of caddisfly species reported from each. Caddisfly regions determined by NMDS ordination (Fig. 7). CN: central, FN: far north, NE: northeastern, NW: northwestern, SE: southeastern, SW: southwestern. Data from Rasmussen and Morse (2023) as well as smaller studies and our own unpublished data. State abbreviations: https://www.fs.usda.gov/database/feis/format.html.

**Figure 2.**
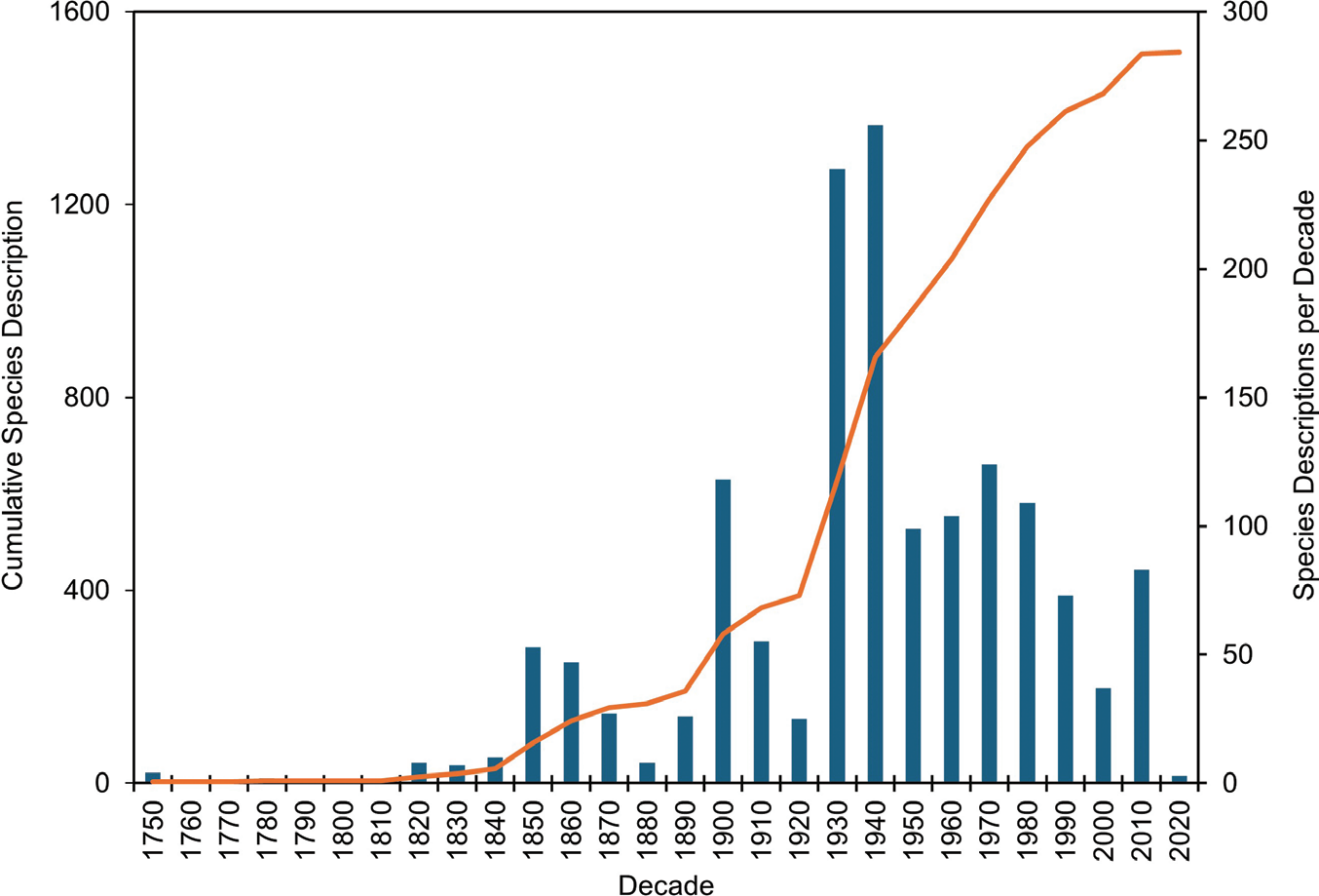
The number of caddisfly species described per decade from Canada and the USA, and the cumulative total from the 1750s to the 2020s.

The adjacent countries of Canada and the United States of America (USA) collectively compose > 90% of the ice-free land area of the Nearctic region (de Moor and Ivanov 2008), encompassing ~20,000,000 km^2^ within their 63 political subunits (hereafter called “states and provinces”) (Fig. 1). While the caddisflies of the two countries have been studied for almost 270 years (Fig. 2), data on species distributions remain coarse and incomplete, and these knowledge gaps limit the ability to answer questions about richness and endemism. Moreover, regional caddisfly assemblages are currently being restructured by local anthropogenic impacts and ongoing climate change, obscuring historical distributions (Murray-Stoker et al. 2019). Such changes, including species extirpations and changes in functional feeding group ecology, have been clearly identified within the northcentral USA where upstream habitat quality is now the primary driver of caddisfly community structure and species richness (Houghton and Holzenthal 2010; Houghton and DeWalt 2021; Houghton and DeWalt 2023).

The caddisfly fauna of Canada and the USA is summarized in the Distributional Checklist of Nearctic Trichoptera (Rasmussen and Morse 2023) and has been the basis for regional (e.g. Houghton et al. 2022) and state and provincial checklists. To date, no study has attempted to analyze or quantify large-scale caddisfly distribution patterns within the two countries. Thus, the objective of this study was to extract data from the Nearctic Checklist and elsewhere to provide the first summary of caddisfly taxonomic work from the past three centuries, identify the most prolific taxonomists, determine regions of high endemicity and coarse patterns of species distributions and richness, and to provide direction for future research within Canada and the USA.

## ﻿Methods

A species presence or absence matrix, including authors and description dates, was generated for all 63 states and provinces within Canada and the USA from the Nearctic Checklist, as well as from recent studies not yet incorporated into the checklist and our own unpublished data. State and province species records that were noted as dubious or erroneous in the Nearctic Checklist were marked as absent. Species considered *nomina dubia* were not included. Maps were generated in ArcGIS Pro (ESRI 2024). Chao1, bootstrap, and first-order jackknife extrapolations of species richness within the region were produced from the package “vegan” (Oksanen et al. 2022) in R v. 2024.09.0+375. Species richness estimates were generated using states and provinces as sample locations and the presence–absence matrix as species incidences within states and provinces. To estimate how the level of taxonomic research in a particular state or province influenced its known species richness, the mean number of citations per species per state or province, based on the Nearctic Checklist, was correlated with the total species richness known per state and province. This relationship was tested for significance using a Spearman rank correlation.

Differences in caddisfly assemblages relative to geography were examined with a non-metric multidimensional scaling (NMDS) ordination using the program PC-ORD v. 7 for Windows (Peck 2016). The data matrix consisted of presence (‘1’) or absence (‘0’) values for each species for each of the 62 analyzed states and provinces. Nunavut was excluded from the analysis since only 15 species are known from that province, and extreme outliers tend to distort the overall ordination (Peck 2016). All species were weighted equally. The NMDS ordination was conducted using the default program settings, 250 randomized runs, and a Jaccard distance measure. A Monte Carlo test was conducted on each determined axis to assess its difference from a random ordination structure (Dexter et al. 2018).

To examine trends in species richness relative to size of state or province and environmental data, seven variables were tested for their collective ability to predict known species richness in the 62 states and provinces using multiple linear regression modeling. The analysis was conducted using Excel for Windows with the Real Statistics add-in (http://ww.real-statistics.com). Nunavut was again excluded from the analysis. Latitude and longitude were determined from the approximate middle of each state or province using Google Earth. Total land area, total freshwater area, and percentage of state or province area composed of freshwater were determined from http://www.census.gov for the United States and http://www.statcan.gc.ca for Canada. Mean temperature and precipitation were determined for each state and province from http://www.currentresults.com. Once a model was produced, its predicted species richness values were compared to reported species richness values for each individual state and province, including Nunavut. This analysis identified states and provinces with reported richness below predicted richness as those for which further research will more likely discover additional species. In addition, the density of species for each state and province was calculated as a number of species divided by the size of the state or province in Mm^2^.

## ﻿Data resources

The data underpinning the analysis reported in this paper are deposited in the Zenodo data repository at https://doi.org/10.5281/zenodo.15176532 (Hogan et al. 2025).

## ﻿Results

**Figure 3.**
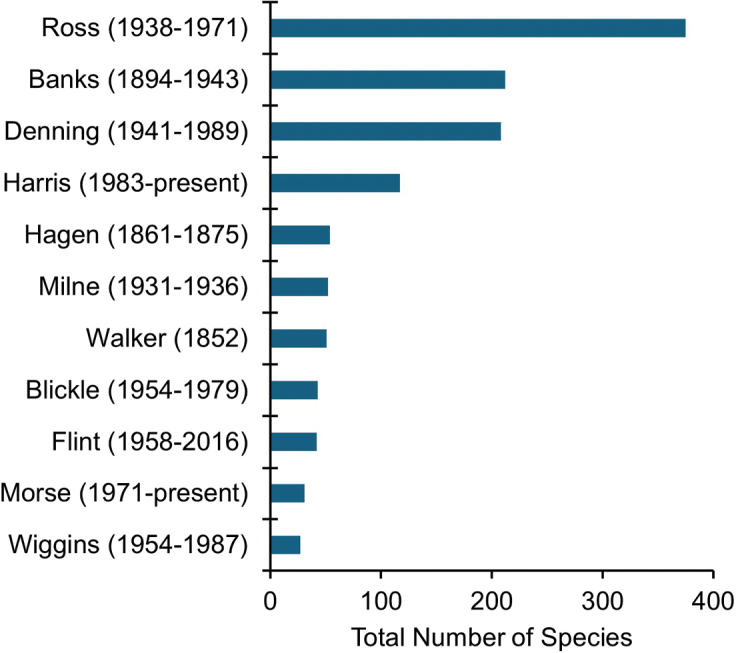
The total number of caddisfly species described by the top 10 most prolific taxonomists from Canada and the USA.

The history of caddisfly taxonomy in Canada and the USA currently spans nearly 270 years (Fig. 2). The first three North American species were described by Linnaeus in 1758 from Sweden and are Holarctic in distribution. The first caddisfly described from a strictly Nearctic distribution, *Glyphopsyche irrorata* (Fabricius) (Limnephilidae), was described 23 years later in 1781. Only 26 species were described during the next 75 years, whereas 127 were described during the 1850s–1870s by Walker and other workers (Fig. 3). Several hundred species were described in the early 1900s by Banks and others. The most prolific period occurred during the 1930s–1940s, when nearly a third of the total fauna (495 species) was described by Ross, Milne, Denning, and others. Ross himself described a quarter (375 species) of the entire fauna from 1938–1971. Species descriptions have averaged around 90 per decade from the 1950s to the 2010s, with about half of the hydroptilid fauna (151 species) described since 1970 by Blickle, Harris, and others (Hogan et al. 2025).

Currently, 1,510 caddisfly species are known from Canada and the USA, representing 28 families and 155 genera (Hogan et al. 2025). A total of 644 species representing 111 genera and 24 families are known from Canada, whereas 1,487 species representing 154 genera and 28 families are known from the USA. The states and provinces with the greatest species richness are Tennessee (384 species), Virginia (383), Alabama (378), and California (366), whereas those with the least are the District of Columbia (55), Rhode Island (27), Prince Edward Island (23), and Nunavut (15) (Fig. 1). The overall mean density of species per area among all states and provinces is 2.77 per Mm^2^, with the highest species densities found in the small northeastern states of District of Columbia (550), Delaware (46), and Connecticut (23) and the lowest in the large far north states and provinces of Alaska (0.16), Northwest Territories (0.10) and Nunavut (0.01) (Hogan et al. 2025).

**Figure 4.**
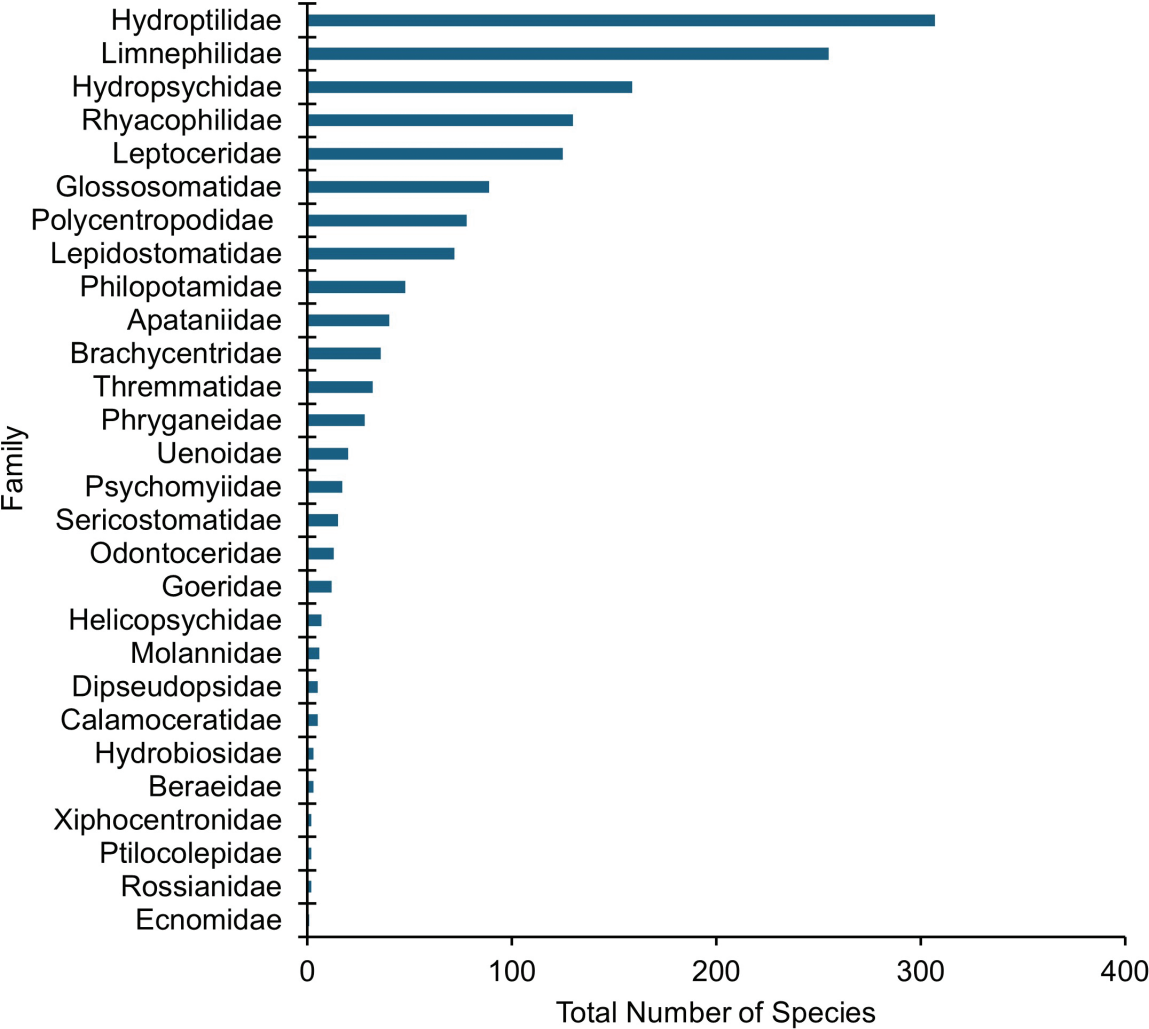
The total number of species for each of the 28 known caddisfly families from Canada and the USA

**Figure 5.**
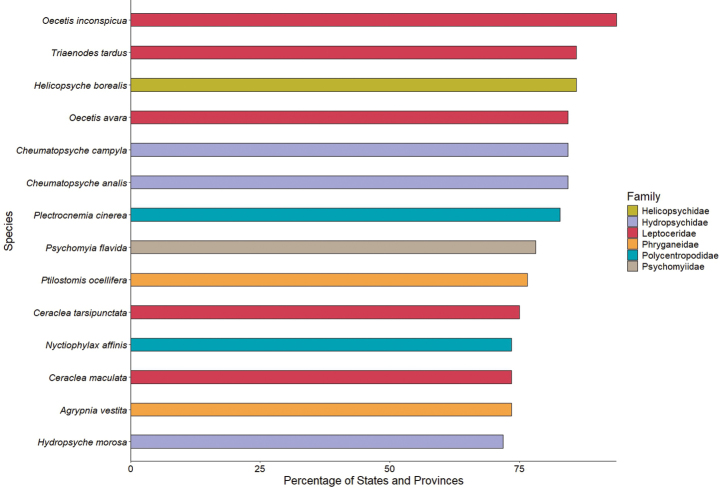
The most widespread caddisfly species, color-coded by family, from Canada and the USA based on the number of states that are known to contain the species.

**Figure 6.**
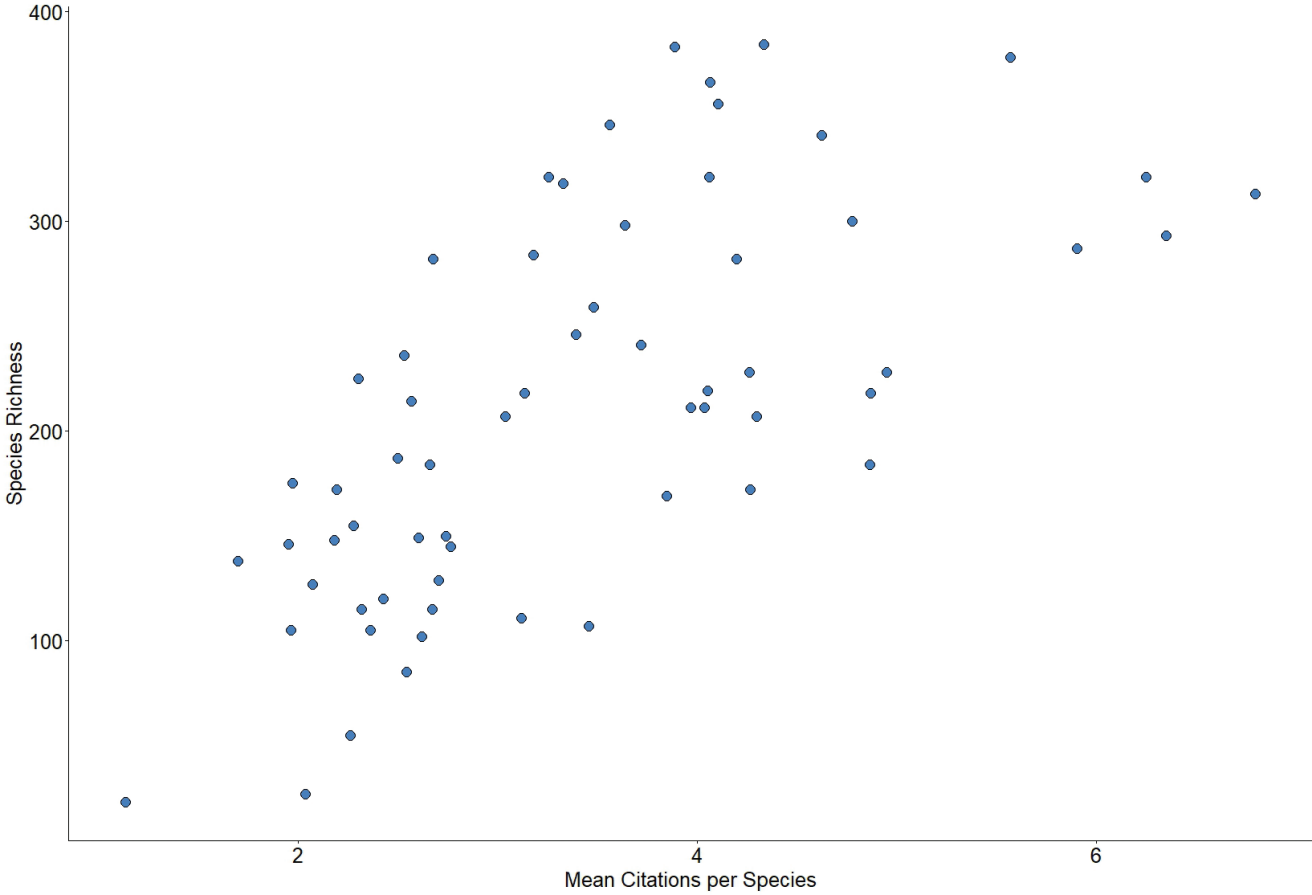
Correlation of species richness and mean citations per species by state and province (*n* = 62, Spearman’s *ρ* = 0.70, *P* < 0.001). Citations that represent the documentation of a particular species in a particular state or province were counted in the Nearctic Checklist.

Among families, Hydroptilidae (307) has the greatest species richness, fo­llowed by Limnephilidae (255), and Hydropsychidae (159) (Fig. 4). The families Beraeidae (3), Hydrobiosidae (3), Ptilocolepidae (2), Rossianidae (2), Xiphocentronidae (2), and Ecnomidae (1) all are represented by fewer than five species in Canada and the USA. *Oecetis inconspicua* (Walker) (Leptoceridae) is the most widespread species, occurring in 60 of the 63 (95%) states and provinces analyzed. *Triaenodes tardus* Milne (Leptoceridae) and *Helicopsyche borealis* (Hagen) (Helicopsychidae) both occur in 55 (87%) states and provinces (Fig. 5). In contrast, 357 species are endemic to a single state or province (Hogan et al. 2025). The state of California (97) has the most endemic species, followed by Oregon (30), Florida (30), Alabama (29), Arizona (29), and Texas (22). All other states and provinces have < 10 endemic species, and 51 of the 63 states and provinces have ≤ 5 (Hogan et al. 2025). Total known species richness within each state and province was positively (Spearman’s *ρ* = 0.70, *P* < 0.001) correlated with the mean number of citations per species of each state and province in the Nearctic Checklist (Fig. 6).

**Figure 7.**
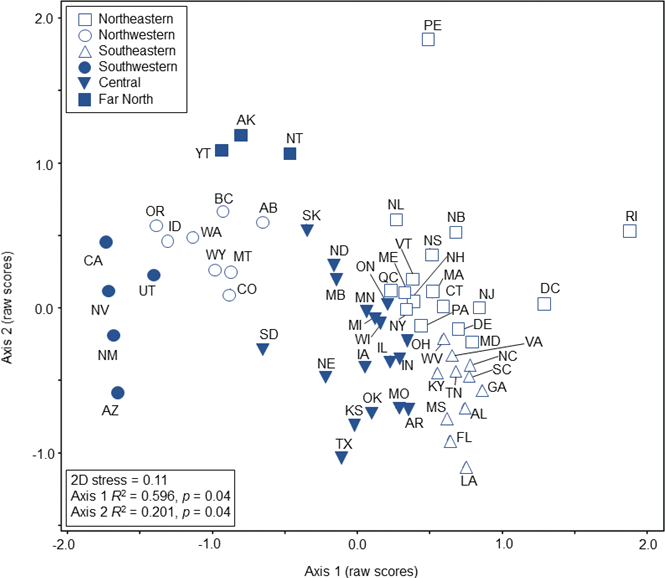
The 62 analyzed states and provinces of Canada and the USA delineated by the results of an NMDS ordination of caddisfly presence or absence per state. The six caddisfly regions determined by ordination results and geographic proximity. Nunavut was excluded from analysis due to only 15 species being reported from it. State abbreviations: https://www.fs.usda.gov/database/feis/format.html.

The NMDS ordination of species presence or absence per state and province produced a two-dimensional solution (Fig. 7). The two determined axes reflected almost 80% of variation within the dataset. Distribution of the 62 states and provinces in ordination space had a high congruence with states and provinces in geographic space, with the three most notable outliers—Rhode Island, Prince Edward Island, and the District of Columbia—also being the states and province of lowest species richness (Fig. 1). The ordination suggested six faunal regions of caddisfly assemblages, corresponding to the central, far north, northeastern, northwestern, southeastern, and southwestern portions of Canada and the USA. The far north, northwestern, and southwestern regions were more distinct from each other than the central, northeastern, and southeastern regions, although there was no geographic overlap between any of the states or provinces in ordination space.

Caddisfly species richness was highest in the southeastern region (684), followed by the central (663), northwestern (608), southwestern (527), northeastern (510), and far north (228) (Table 1). The southeastern region had the highest species richness relative to area and per state. The species richness per area of the southeastern region was especially noteworthy, as it was > 1.5× greater than any other region and > 10× higher than the far north region.

**Table 1.** Summary statistics for the six determined caddisfly regions (Fig. 7) of Canada and the USA.

Region	Area (Mm^2^)	Total species	Species per area	Mean species per state
Southeastern	1327	684	0.52	276.4
Central	5528	663	0.12	200.4
Northwestern	3275	608	0.19	245.7
Southwestern	1554	527	0.34	202.0
Northeastern	2638	510	0.19	182.6
Far North	4750	228	0.05	107.3

**Figure 8.**
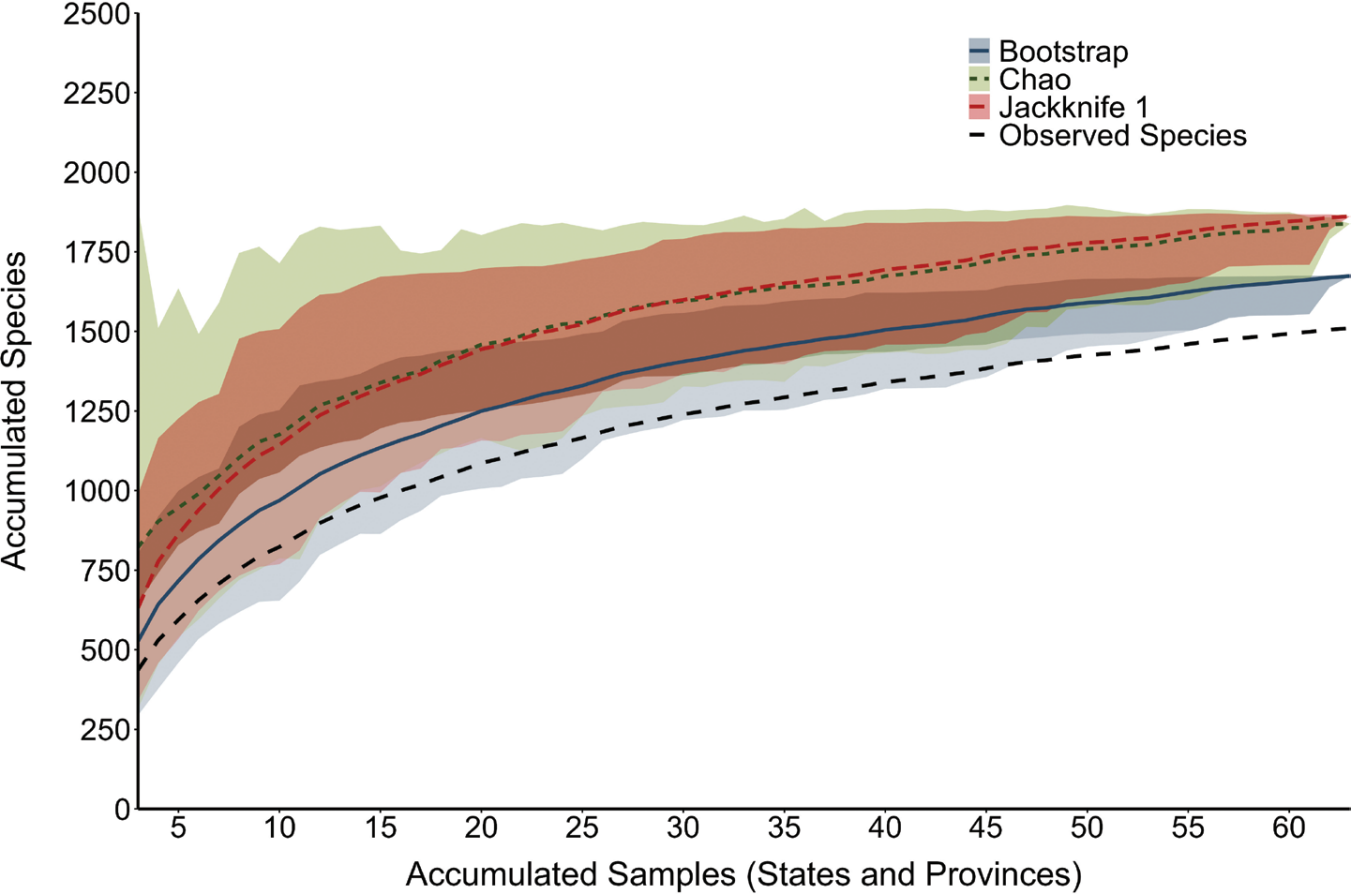
Estimations of total caddisfly species richness for Canada and the USA based on three different estimators.

A multiple linear regression analysis combining all seven variables produced a significant model (*P* = 0.04) with low predictive power (*R*^2^ = 0.12) (Table 2). Species richness increased with increasing temperature and precipitation and decreased with increasing latitude. Comparing reported species richness to species richness predicted by the model suggested 17 states and provinces with ≥ 50 species remaining to be found in each (Table 3). Estimates of total species richness for the entire study area ranged from 1,674 (±72) to 1,861 (±117) from the bootstrap and first-order jackknife methods respectively (Fig. 8).

**Table 2.** Results of a multiple linear regression model of the combined ability of seven variables to predict the known number of caddisfly species within each of the 62 tested states and provinces. Nunavut excluded due to having only 15 species reported to date. Overall model *R*^2^ = 0.12, *P* < 0.043. VIF: variance inflation factor.

Variable	Coefficient	SE	*T*-statistic	*P*	VIF
Intercept	857.93	268.09	3.20	0.002	
Mean latitude	−19.23	6.22	−3.09	0.003	19.46
Mean precipitation	3.58	1.43	2.50	0.015	2.67
Mean temperature	20.17	8.20	2.46	0.017	16.98
Mean longitude	−2.12	1.03	−2.05	0.045	3.25
Total water area	0.00	0.00	0.65	0.516	7.90
Total land area	0.00	0.00	0.40	0.691	8.46
Percent water area	−37.93	193.35	−0.20	0.845	2.06

**Table 3.** The 17 states and provinces, and their land and freshwater areas, that are predicted to contain at least 50 more caddisfly species based on difference in reported species richness relative to the model generated in Table 2.

State	Reported richness	Predicted richness	Additional species	Land area (Mm^2^)	Water area (Mm^2^)
Nunavut	15	200	185	1936.1	157.08
Louisiana	105	290	185	111.9	23.76
Prince Edward Island	24	168	144	5.7	0.54
New Brunswick	105	248	143	71.5	1.46
Rhode Island	27	168	141	2.7	1.32
Mississippi	154	283	129	121.5	3.91
Alaska	150	278	128	1478.0	245.38
District of Columbia	55	174	119	0.2	0.02
New Mexico	118	236	118	314.2	0.76
Nebraska	102	183	81	199.0	1.36
Nova Scotia	149	228	79	53.3	1.95
Nevada	140	219	79	284.3	2.05
Kansas	108	185	77	211.8	1.35
Iowa	127	204	77	144.7	1.08
South Dakota	84	155	71	196.4	3.40
Northwest Territories	114	165	51	1183.1	163.02
Arkansas	207	257	50	134.8	2.96

## ﻿Discussion

The congruence of state and province species assemblages with geographic location was noteworthy and probably due to a combination of natural factors. Both latitude and longitude are known to affect caddisfly assemblages (Houghton 2004; Blinn and Ruiter 2013; Shah et al. 2014), and some assemblage differences in the study area certainly reflected species replacement over geographic distance. Differences in environmental factors such as temperature, precipitation, and habitat heterogeneity also undoubtedly affected the composition and richness of species assemblages. The high species richness of the southeastern region, for example, was likely due to its warm and wet climate, coupled with the elevational and habitat differences associated with the Appalachian Mountain range. Similarly, the state of California probably had the most endemic species due to its diversity of unique and isolated environments, including both the highest mountain and lowest desert in the continental USA. Conversely, the arid and homogenous prairie environments of the western central region are less conducive to habitat and species diversity (McNeely 2003); therefore, nearly all prairie states had low caddisfly richness. Similarly, the far north region had less than half the species richness of other regions despite its large land area with heterogenous and undisturbed habitats due largely to its cold climate and short growing season (Scott et al. 2011). Size of the individual states and provinces was also important as, unsurprisingly, the states and provinces with greatest species density were all of very small area.

The distinct faunistic differences between the eastern and western states and provinces of our study area also reflect their glacial histories. Complex drivers of species distributions such as historical climatic shifts of the Pleistocene contributed to the evolution of narrow range endemics south of the glacial maxima through geographic isolation within glacial refugia (Ross 1956; Chuluunbat et al. 2010). Likewise, glacial history, particularly the advance and retreat of the Cordilleran and Laurentide ice sheets following the Last Glacial Maximum (~21 kya) structured contemporaneous distributions as species dispersed northwards along the glacier’s retreating edge. Further dissimilarity between the eastern and western regional fauna is due in part to the biogeographical affinities of the far north and northwestern region with the eastern Palearctic realm and the southwestern region with the Neotropical realm, leading to the presence of multiple families and genera that are not found in the central, northeastern, and southeastern regions.

Collecting effort also affected known richness of caddisfly assemblages. States and provinces with a long and robust taxonomic history, such as Alabama (Harris et al. 1991), Kentucky (Floyd et al. 2012), Tennessee (Etnier et al. 1998), and Virginia (Flint et al. 2004, 2008) in the southeastern region, Illinois (Ross 1944; Houghton et al. 2022), Indiana (Waltz and McCafferty 1982, 1983; Houghton and DeWalt 2024), Michigan (Houghton et al. 2018), Minnesota (Houghton 2012), Ohio (Armitage et al. 2011), and Wisconsin (Vorhies 1908; Longridge and Hilsenhoff 1973) in the central, California (Mendez et al. 2019) in the southwestern, and New York (Betten 1934; Myers et al. 2011), Pennsylvania (Masteller and Flint 1992), and Quebec (Roy and Harper 1979) in the northeastern all had some of the highest species richness of their respective regions. Conversely, states and provinces such as Nunavut, Georgia, and South Dakota do not yet have statewide or provincial checklists compiled and all have low richness for their regions. The known caddisfly faunas of Maryland (Hogan unpublished data) and Nebraska (Houghton and Peterson 2025) have both increased markedly with recent collecting, suggesting that many more records remain to be found in other states and provinces with low effort.

Integrative taxonomic review may also increase state, province, and regional species richness. Differences in morphological and genetic characters suggest that the most widely distributed species in our study area, *O. inconspicua*, probably represents a species complex (Floyd 1995; Zhou et al. 2011). Genetic evidence questioning monophyly is not uncommon for caddisfly species, suggesting the necessity of species or genus revisions (Jackson and Resh 1992, 1998; Zhou et al. 2011; Harvey et al. 2012). However, the inadequacy of current resources designated toward taxonomy, the “taxonomic impediment”, remains a major obstacle to describing and quantifying diversity (Löbl et al. 2023). Allocating more resources toward training new taxonomists with genetic or genomic experience will help evaluate current species designations and address cryptic species. This, in conjunction with the increasing capability to obtain high quality genomic material from historical specimens through minimally destructive practices (Heckenhauer et al. 2023), may provide the basis of future caddisfly species descriptions and delimitations.

The 163–351 total species estimated to be still undiscovered throughout the entire study area represents 11–23% of the total fauna. Sheffield et al. (2019) estimated 129–181 species remain to be documented in Canada alone. Of the incidence-based, non-parametric estimators of species richness, the first-order jackknife estimator performs consistently better than the Chao or bootstrap estimators, however, at larger sample sizes, the bootstrap estimator produces more accurate estimates (Hortal et al. 2006). The jackknife and Chao estimators can provide inflated estimates of species richness when many rare species are found within the community, whereas the bootstrap estimator provides a conservative estimate of species richness independent of species’ rarity (Walther and Morand 1998). Each estimator applied indicates that additional species remain to be documented throughout the study region. However, 25% of all species present in Canada and the USA are endemic to single states or provinces, indicating that the jackknife and Chao estimates may be influenced by the number of rare species. Therefore, the bootstrap estimate of 1,674 (±72) species may be the most accurate.

Future caddisfly work in Canada and the USA should include greater effort in under-collected states and provinces. While our model of predicted species richness per state and province was based on coarse data and not particularly robust in its confidence, it still clearly indicated states and provinces that should be future priorities for caddisfly research. Only 15 species have been reported from Nunavut in the far north region, despite having the largest land area of any state or province. Even when excluding its extreme northern islands, Nunavut still has more than two-thirds as much land area as the entire southeastern region, and there are undoubtedly many records and undescribed species remaining in its mostly undisturbed lakes and rivers. Georgia, Louisiana, and Mississippi in the southeastern region all contain far fewer species than neighboring Alabama, despite having similar size, location, temperature, and precipitation. Similarly, New Brunswick in the northeastern region has almost the same land area as adjacent Maine yet contains one-third of the known caddisfly richness. Most of the central region prairie states, such as Iowa, Kansas, Nebraska, and South Dakota probably contain more species than reported. Unfortunately, those states are also some of the most disturbed, primarily by agriculture (Houghton 2021), and have probably already experienced species extirpations (Houghton and DeWalt 2023). States and provinces such as Prince Edward Island, Rhode Island, and the District of Columbia are also predicted to have greater than reported species richness, but their small size may explain the lower richness and—especially for the last two—high levels of urbanization may have already lowered the original total.

Given the modest faunal knowledge for many states and provinces, field methodology should be considered carefully in planning future study designs to ensure time and resources are utilized efficiently. Current standard practices, such as ultra-violet lights, are biased toward sampling nocturnal-flying adults of late spring and summer emergent species. Long-term passive and active sampling methods such as flight-intercept traps, laboratory rearing of larval material, or emergence traps, should be considered to inventory regional fauna. A quantitative comparison of the advantages and disadvantages between each passive and active collecting technique in addition to describing differences between species diversity, biomass, and sex ratios of specimens collected will provide strong direction for future faunistic studies (Pereira et al. 2021). While additional collecting effort will fill knowledge gaps, future faunistic summaries should also include ecoregion data and fine-scale distribution patterns of species, rather than using merely the coarse scale of political unit. Review, digitization, and data publication of specimens archived in natural history museums will help clarify individual patterns and determine both contemporary and historical distributions. Genetic characterization of species associated with vouchered specimens in natural history museums through DNA barcoding or genomic sequencing will further advance the rate of species descriptions and unveil interspecific evolutionary relationships, particularly in taxa where cryptic species are suspected. Unfortunately, taxonomic expertise comes only after years of training, and the number of trained insect taxonomists has been in decline for several decades (Moulton 2004), leading to the challenge of elucidating distribution information for a fauna already in decline (Houghton and DeWalt 2023). One solution to this problem may be in community fieldwork (Murray-Stoker and McCauley 2023), thus freeing time for taxonomic experts to identify samples and describe species diversity. Regardless of the difficulties and despite nearly 270 years of effort, many new caddisfly species and even more distribution records undoubtedly remain to be discovered in Canada and the USA.

## ﻿Additional information

### Author ORCIDs

Phillip N. Hogan https://orcid.org/0000-0002-6546-272X

David C. Houghton https://orcid.org/0000-0001-6946-4864

Kelly Murray-Stoker https://orcid.org/0000-0001-7153-8092

R. Edward Dewalt https://orcid.org/0000-0001-9985-9250

Andrew K. Rasmussen https://orcid.org/0009-0005-9549-289X

John C. Morse https://orcid.org/0000-0003-3187-4045

### Data availability

All of the data that support the findings of this study are available in the main text or Supplementary Information.
